# Remineralization of Artificial Caries in Primary Teeth by Grape Seed Extract: An *In Vitro* Study

**DOI:** 10.5681/joddd.2013.033

**Published:** 2013-12-18

**Authors:** Mahkameh Mirkarimi, Solmauz Eskandarion, Majid Bargrizan, Abbas Delazar, Mohammad Javad Kharazifard

**Affiliations:** ^1^Assistant Professor, Department of Pediatric dentistry, Children and Adolescent Health Research Center, Zahedan University of Medical Sciences, Za-hedan, Iran; ^2^Assistant Professor, Department of Dental Materials, Faculty of Dentistry, Shahid Beheshti University of Medical Sciences, Tehran, Iran; ^3^Associate Professor, Department of Pediatric Dentistry, Faculty of Dentistry, Shahid Beheshti University of Medical Sciences, Tehran, Iran; ^4^Professor of Faculty of Pharmacy and Drug Applied Research Center, Tabriz University of Medical Sciences, Tabriz, Iran.; ^5^Phd Candidate of Epidemiology Department of Epidemiology and Biostatistics, School of public health, Tehran University of Medical Sciences, Tehran, Iran

**Keywords:** Caries, enamel, grape seed extract, microhardness

## Abstract

***Background and aims.*** Promoting remineralization is the ultimate goal of clinical prevention of caries lesion. The present* in vitro *study aimed to investigate the effect of grape seed extract (GSE) on artificial enamel caries in primary human teeth.

***Materials and methods.*** Seventeen human sound primary incisors were sectioned mesiodistally. The tooth slices were placed in a demineralizing solution for 96 hours at 37ºC and 50% relative humidity to create lesions. The demineralized fragments of each tooth were randomly divided into two case (immersed in GSE solution in phosphate buffer for 8 days) and control (immersed in distilled water) groups. The samples were subsequently evaluated using a scanning electron microscope and a micro-hardness tester. Data were analyzed using independent t-test.

***Results.*** The mean ± SD micro-hardness values for the case and control groups were 358.6±83.42 and 296.51± 69.41, respectively. Grape seed extract significantly increased the micro-hardness of the lesions (P=0.03). The morphology of GSE treated enamel was clearly different from that in the control group, and there were deposits of scaffolding insoluble complexes on the enamel surface.

***Conclusion.*** GSE enhanced the remineralization process of artificial enamel lesions of primary teeth, and thus, might be considered an effective natural agent in non-invasive dentistry.

## Introduction

Natural products have been used in medicines for thousands of years and are promising sources for novel therapeutic agents,^1^ especially in oral diseases such as dental caries.^2,3^

Teeth are constantly going through cycles of demineralization and remineralization. The ultimate goal of clinical intervention is the preservation of tooth structure and prevention of lesion progression to the point where restoration is required. While fluoride is an established agent in promoting remineralization and inhibiting demineralization of enamel, other agents exist for creating favorable remineralization conditions in the oral cavity like Casein phosphopeptide amorphous calcium phosphate (CPP-ACP) complex,^4^and ß-tricalcium phosphate (ß-TCP).^5^ Grape seed extract (GSE) is a rich source of proanthocyanidin (PA), mainly composed of monomeric catechin and epicatechin, gallic acid and polymeric and oligomeric procyanidins.^6^

Proanthocyanidins has been reported to strengthen collagen-based tissues by increasing collagen cross-links. There is evidence that PA increases collagen synthesis and accelerates the conversion of soluble collagen to insoluble collagen.^3^ PA has proved safe in various clinical applications and has been used as dietary supplements.^7,8^

It has been shown that GSE positively affects the remineralization process of root caries.^2^ Since collagen can serve as a substrate for apatite formation,^11,12^ the present study was designed to assess whether GSE, mainly consisting of PA, can effectively influence the remineralization of artificial caries in human primary teeth.

## Materials and Methods

###  Preparation of Grape (Vitis vinifera L.) Seed Extract

Ground grape seeds (100 g) were extracted with ethanol to water ratio of 70:30, v/v, by maceration method. The extracts were then filtered.

### Determination of Total Phenol Content (TPC)

The total phenol content of the grape seed extract was determined by the Folin-Ciocalteu method. One mL of GSE solution in aceton/water (6/4) was transferred to a test tube and then mixed thoroughly with 0.2 mL of Folin-Ciocalteu reagent. After mixing for 3 min, 1 mL of 2% (w/v) sodium carbonate was added. The mixtures were agitated with a vortex mixer and then kept in dark for 30 min, after which they were centrifuged at 12000 g for 5 min. The absorbance of the extracts and a prepared blank were measured at 750 nm using a spectrophotometer. The measurements were compared to a standard curve of prepared gallic acid solution and expressed as grams of gallic acid equivalents (GAE) per 100 grams of the extract, which was determined from known concentrations of gallic acid standard prepared similarly. The TPC of GSE was 70 g GAE/100 g.

### Specimen Preparation

Seventeen extracted sound human primary incisors were selected. They were thoroughly cleaned of organic debris and stored in 0.5% chloramin solution for 24 h and then immersed in distilled water (grade 3, ISO 3696). The teeth were sectioned mesiodistally by a low-speed diamond saw cooled by water. The sectioned surfaces of the teeth were covered with an acid resistant nail varnish. The tooth slices were stored at 4ºCprior to use (according to ISO/TC 11405).

### Lesion Formation and Remineralization Test

The tooth slices were placed in a demineralizing solution (2.2 mM of CaCl_2_.2H_2_O, 2.2 mM of KH_2_PO_2_,45 mM of acetate, pH = 4.6) for 96 h at 37ºC and 50% relative humidity to create lesions. Subsequently, the fragments were rinsed thoroughly with deionized water. The demineralized fragments of each tooth were randomly divided in two groups. One of the sections was immersed in distilled water (control group) and the other section was immersed in GSE solution (12.5% w/v) in phosphate buffer (3.4 gr of KH_2_PO_4_, 782 mg of NaOH) for 8 days (case group). All solutions were freshly made on a daily basis prior to use.

### Micro-hardness Test

To test micro-hardness, 15 case and 15 control samples were rinsed with deionized water and embedded in epoxy resin for micro-hardness evaluation; the two remaining samples were used for scanning electron microscope analysis. The embedded samples in epoxy resin were grounded flat with water-cooled discs (60 to 3000 grits of SiC papers, Matador, Germany) and polished with 1-µm Al_2_O_3_ felt papers (Struers, Denmark). The surface micro-hardness of the enamel was measured using a micro-hardness tester (Five HMV 2000, Shimadzu Corporation, Tokoyo, Japan) with a Knoop diamond under a load of 50 gr/10s. Three indentations were made on each specimen and the average values were calculated. The average values for the case and control sections of each tooth were compared by independent t-test.

### Scanning Electron Microscope Analysis

The two remaining samples were gold-coated for scanning electron microscope evaluation. The morphology of enamel surfaces was evaluated under a scanning electron microscope (SEM, Philips, XL30 Scanning Microscope, Philips, Netherlands). An intact enamel surface of a primary incisor tooth was also observed for better comparison of the samples.

## Results

**Figure 1.**
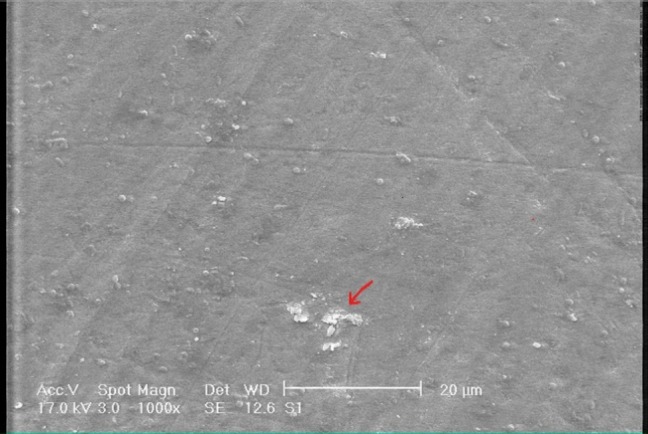
Sound enamel structure with a smooth sur-face (×1000 magnification) and spherical particles on the surface (arrow).

**Figure 2.**
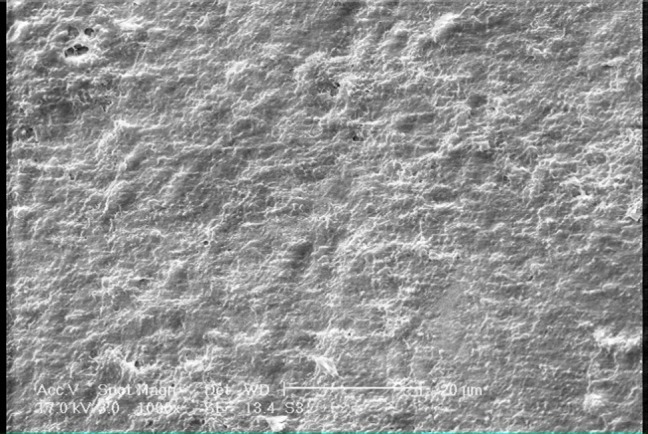
Demineralized enamel showing a rough surface with significant porosities (×1000 magnification).

Figure 1shows the SEM photomicrograph of sound enamel (×1000 magnification), revealing an orderly smooth appearance. There are also some spherical particles on the surface.Figure 2shows the photomicrograph of demineralized enamel (×1000 magnification) before treatment; the enamel surface is rough and disorganized with significant porosities. In Figure 3Ashowing the demineralized enamel exposed to GSE (×1000 magnification), there are coating depositions of some insoluble complexes on the enamel surface. The reaction products of GSE are seen as amorphous clumps. Spherical globular agglomerates were observed on the surface of the enamel, with varying sizes from place to place. Figure 3Bpresents the previous photomicrograph at ×2500 magnification.

Figure 3. (A) The appearance of demineralized enamel treated with grape seed extract (×1000 magnification) showing scaffolding deposits (arrow). (B) Spherical particles are obvious under ×2500 magnification (ar-row).

**A:**
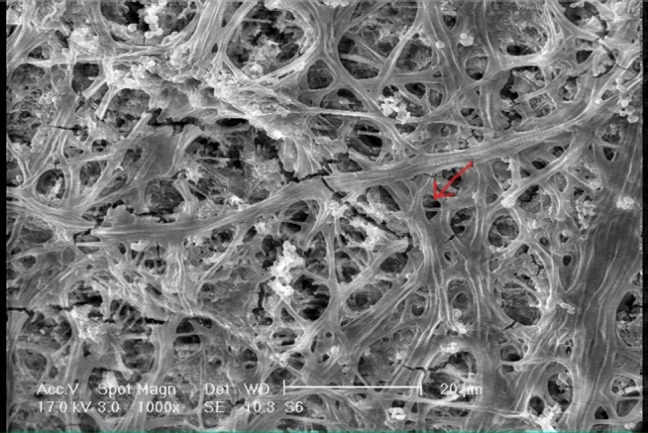

**B:**
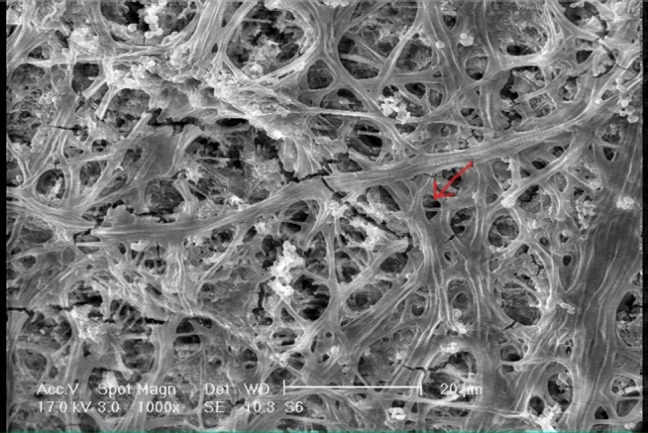

**Figure 4.**
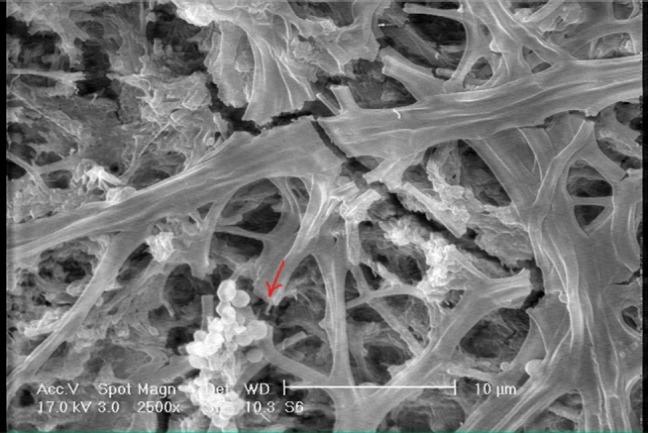
Micro-hardness values of the case and con-trol groups as compared by the independent *t*-test.

Table 1 demonstrates the mean micro-hardness values (±SD) of the two groups. Independent t-test revealed statistically significant differences between the two groups (Figure 4). Samples treated with GSE had significantly higher micro-hardness values compared with the control group (P=0.03).

**Table 1:** Enamel micro hardness values of the case and control groups

	Group	N	Mean	Std. Deviation	Std. Error Mean	95% Confidence Interval of the difference
Hardness	Case	15	358.6000	83.42426	21.54005	4.6-119.4
	Control	15	296.5111	69.41592	17.92311	

## Discussion

Grape seed extract has recently been advocated for its beneficial antioxidant, antibacterial and free radical scavenging properties.^11,12^ The biologically active constituents of GSE are polyphenols, mainly proanthocyanidins, which are condensed tannin; it represents a variety of polymers of flavan-3-ol such as catechin and epicatechin.^13^ The GSE used in this study consisted of 90 percent proanthocyanidin, measured by Vanillin HCl assay technique. Some studies have evaluated the effect of GSE on demineralized dentin,^2^ but the effect of this agent on the remineralization of enamel defects is not well-understood. According to Cheng et al,^14^ gallic acid, one of the major constituents of grape seed extract and galla chinensis, facilitates mineral deposition, predominately on the surface layer. In the SEM analysis of the present study, discontinuous and broken enamel crystals were visible after the demineralization process. After treating with GSE, there were scaffolding deposits on the enamel surface with cluster-like structures resembling remineralization process initiation. Spherical particles were also visible on sound enamel surface and, to a more extent, on treated enamel surface, and according to Olmez et al,^15^ these might be CaF_2_ deposits which are more resistant to demineralization process. It should be pointed out that the fluoride concentration in the GSE was about 0.01 ppm as measured by a fluoride electrode (ThermoFisher Scientific Orion Ionplus Fluoride Elctrd, 9609BNWP). It is not clear whether this low concentration of fluoride in a solution can result in the formation of CaF_2_ spherical deposits, a hypothesis that is yet to be confirmed. On the other hand, GSE significantly increased micro-hardness of carious lesions compared to the control group. In this aspect, GSE may be comparable with other materials like CPP that can obviously increase the surface hardness of the enamel.^16^ On the other hand, some authors have proposed that CPP-ACP deposits a high concentration of ACP in close proximity to the tooth surface,^4^ and, as mentioned before, GSE deposits were found on the enamel surface in the present study. According to Xie et al,^2^ GSE positively affects the demineralization and/or remineralization process of artificial root caries. Berden Russo et al^17^ demonstrated that GSE, as a collagen cross-linker, increased the stiffness of demineralized dentin. Furthermore, GSE has been shown to improve the ultimate tensile strength of demineralized dentin.^18^ Al-Ammor et al^19^ reported that the chemical modification of dentin matrix promoted by GSE resulted in increased bond strength. In this context, GSE might contribute to mineral deposition on the superficial layer of the lesion by formation of insoluble complexes when mixed with bufferic phosphate solution; in addition, GSE might interact with proteins to induce cross-links by four different mechanisms: covalent interaction, ionic interaction, hydrogen bonding interaction and hydrophobic interaction.^2,19,20^ Proline-rich proteins like collagen have an extremely high affinity for PA-based components, forming a proline–PA complex.^19,21^ Although traditionally mature dental enamel is considered to be free of collagen, Acol et al^22^ showed that this is not the case and type I collagen is found in enamel; however, the concentration of collagen in enamel was considerably lower as compared to that in dentin. Furthermore, Felszeghy et al^23^23 found that type X collagen is one of the candidate molecules present in the enamel matrix, which might be involved in mineralization of enamel. Considering these findings, it is not surprising to find exogenous collagen cross-links produced by the positive effects of remineralization of enamel defects by GSE in the present study in SEM micrographs. It should be pointed out that the terminal carboxyl groups and amine groups mainly contribute to the absorption of collagen peptides to the hydroxyapatite surfaces. In addition, the –OH and positively charged –NH3^+^ groups of peptides in particular bind strongly to the surfaces and their presence should therefore promote hydroxyapatite growth.^24^

Based on data obtained in this *in vitro* study, it may be proposed that GSE promotes the remineralization process of artificial carious lesions in the enamel of primary teeth. It should be noted that the oral cavity is different from an experimental environment and the obtained results should further be evaluated by *in vivo *studies.

## Conclusion

Grape seed extract has positive effects on the remineralization process of artificial caries lesions of the enamel in human primary teeth *in vitro*. This solution might be considered an effective natural agent for non-invasive therapy of carious lesions in children.
